# Adalimumab-Responsive Refractory Sarcoidosis Following Multiple Eyebrow Tattoos: A Case Report

**Published:** 2017

**Authors:** Alireza Mirzaei, Mohamad Mehdi Joharimoghadam, Mozhdeh Zabihiyeganeh

**Affiliations:** 1 Bone and Joint Reconstruction Research Center, Shafa Orthopedic Hospital, Iran University of Medical Sciences, Tehran Iran; 2 Faculty of Medicine, Iran University of Medical Sciences, Tehran, Iran.

**Keywords:** Refractory sarcoidosis, Eyebrow, Adalimumab

## Abstract

Sarcoidosis is a granulomatous disease that can involve multiple organs including the lungs, eyes, nerves, and skin. Cosmetic tattooing can be a predisposing factor for sarcoidosis, and its incidence is likely to increase along with its popularity.

A 47-year-old woman with symptoms of fever and polyarthritis along with erythema nodosum lesions on the legs, swollen eyebrows, and a history of multiple eyebrow tattooing was referred to our center. Since the signs and symptoms were positive for Löfgren’s syndrome triad including erythema nodosum, bilateral hilar lymphadenopathy, and polyarthritis, a diagnosis of acute sarcoidosis was made, and treatment was started accordingly. The patient was unresponsive to routine therapeutic agents used for sarcoidosis; however, she successfully responded to adalimumab.

Our case indicates that systemic sarcoidosis could develop as a reaction to cosmetic tattooing, which might be refractory to conventional therapeutic agents including corticosteroids and non-biologic disease-modifying anti-rheumatic drugs, while tumor necrosis factor antagonists such as adalimumab, could lead to disease remission.

## INTRODUCTION

Sarcoidosis is a granulomatous disease that affects multiple organs including the lungs, eyes, nerves, and skin. Cosmetic tattooing has been frequently cited as a predisposing factor for sarcoidosis. Foreign materials such as pigments in the tattoo ink, stimulate the body’s immune system in a genetically susceptible person. Chronic low-grade exposure of the immune system to repeated cosmetic tattooing can lead to systematized granulomatous hypersensitivity, with a long latency period (1–3). Similar to other cases of hypersensitivity, avoiding the causative antigen in this case may result in remission of symptoms (2). However, in some cases, such as cosmetic tattooing, exposure to the antigen cannot be avoided, and hence, more invasive approaches are necessary.

There is no consensus regarding the indication and duration of the treatment for sarcoidosis. Treatment is usually recommended in patients with aggravated respiratory symptoms, especially shortness of breath and cough. Other reasons for treatment include signs of reduced lung function as determined through pulmonary function tests, or difficulty in performing daily activities due to fever, weakness, fatigue, joint pain, nervous system changes, disfiguring skin disease, or disease affecting the upper airway.

Although the disease remits spontaneously in most patients, 10 to 30% of patients develop chronic disease that could be refractory to multiple lines of treatment (4). Although there is minimal evidence-based data for pharmacologic management of sarcoidosis, a stepwise treatment approach is usually followed, ranging from corticosteroids for chronic cases to anti-tumor necrosis factor (TNF) therapy for refractory cases (5).

Here, we present the case of a 47-year-old woman with refractory systemic sarcoidosis that was induced by eyebrow tattooing and was successfully treated with adalimumab, a recombinant human IgG1 monoclonal antibody that binds specifically to TNF-alpha.

## CASE SUMMARIES

A 47- year-old woman with no significant medical history was referred to our center with pain in the interphalangeal joints of the hands and the knees and ankles, erythematous nodules on shins, and swollen eyebrows. The symptoms had appeared 2 months before the patient’s referral.

**Figure 1.**
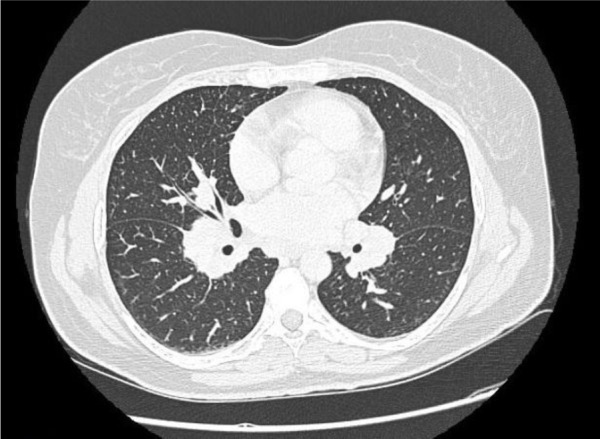
Spinal CT scan of Thorax with IV contrast showing systemic hilar and mediastinal adenopathies with subtle reticulonodular lungs infiltration compatible with sarcoidosis.

On clinical evaluation, polyarthritis along with symptoms of erythema nodosum-like nodules and low-grade fever was detected. Distinct red papules were visible above the eyebrows (Figure 1).

Results of routine hematological and biochemical tests including serum calcium were normal. Immunologic tests including anti-nuclear antibody (ANA), rheumatoid factor (RF), and tuberculin test were negative. Additionally, the erythrocyte sedimentation rate (ESR) was 51 mm/hr (normal range: 0–29 mm/hr for women), angiotensin-converting enzyme (ACE) level was 73 U/L (normal: less than 40 U/L), and C-reactive protein (CRP) level was 48 mg/L (normal: less than 10 mg/L). The patient reported a history of multiple tattooing over the eyebrows, and the last tattooing was performed 4 months before her present symptoms manifested.

Considering the presence of erythema nodosum and bilateral ankle arthritis, computed tomographic scan (CT) of thorax was performed, which showed bilateral hilar adenopathy with reticulonodular lesions in lower lobes of the lung (Figure 1). The laboratory test of tuberculosis performed via direct examination and culture of the sputum was negative. The diagnosis of Löfgren’s syndrome, an acute form of sarcoidosis was confirmed based on the presence of the triad of erythema nodosum, bilateral hilar lymphadenopathy, and polyarthritis (6).

Considering a 99.95% positive predictive value of Löfgren’s syndrome for the diagnosis of sarcoidosis (6), biopsy was not advised.

Prednisolone 30 mg/day along with azathioprine 100 mg/day as a steroid-sparing agent were administered to the patient. After a follow-up period of 6 weeks, improvement in joint and cutaneous symptoms was observed. However, a four-fold increase in the liver enzymes led to the discontinuation of azathioprine. Subsequently, the prednisolone dose was tapered to 2.5 mg weekly. However, at the next follow-up one month later, due to increased inflammation and erythema of the eyebrow lesion along with recurrence of previous symptoms, the dose of prednisolone was increased to 50 mg/day and intralesional corticosteroid injection was administered as well. Methotrexate (MTX), 15 mg injection per week, was also added to the treatment as an alternative steroid-sparing agent. However, no improvement was observed after 8 weeks.

**Figure 2.**
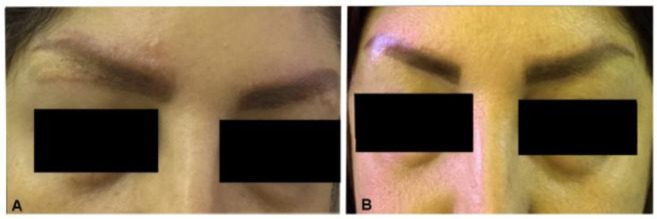
(A) Cutaneous eyebrow granulomatosis following multiple eyebrow tattooing; (B) Disappearance of eyebrow inflammation and erythematous papules after Adalimumab administration

Eventually, a biopsy of the eyebrow was recommended. However, due to the fear of a post-biopsy scar, the patient refused to undergo biopsy. Following the patient’s complaint of increased appetite and weight gain but no improvement in symptoms, treatment with subcutaneous adalimumab 40 mg (one ampoule) every 2 weeks along with 10 mg prednisolone was started. The inflammation and erythematous papules disappeared in 2 weeks after the first injection (Figure 2). A follow-up chest CT after 4 weeks showed no significant abnormality. Subsequently, prednisolone was tapered and withdrawn over a period of 3 months, and adalimumab was continued as monotherapy.

Despite the early evidences of adalimumab effectiveness in sarcoidosis treatment, there is no standard protocol for such intervention. We administered two ampoules of adalimumab subcutaneously every month (80 mg in total), as suggested for rheumatoid arthritis (7).

After 2 months of adalimumab treatment, increasing intervals between the injections from 2 weeks to 3 weeks (one 40 mg adalimumab ampoule per 3 weeks) led to the re-emergence of symptoms, which required a subsequent interval reduction to 2 weeks again. After 9 months of follow-up, the patient is still receiving adalimumab as monotherapy, and no sign of disease recurrence has been observed.

## DISCUSSION

Systemic sarcoidosis following tattooing of different parts of the body has been often reported (3). Various therapeutic approaches, including surgical removal of lesions with or without pharmacologic treatment, have been proposed. However, no standard therapeutic option is suggested due to minimal evidence-based data (5). Besides, treatment of sarcoidosis is directed by manifestations of the disease. Initial treatment for the cutaneous form is usually with topical corticosteroids. Systemic corticosteroid therapy is administered to the cases of systemic sarcoidosis. For the treatment of refractory sarcoidosis, corticosteroids, along with a steroid-sparing agent such as MTX, are recommended. TNF antagonists were originally indicated for the treatment of rheumatoid arthritis. They act by interfering with the production of certain proteins involved in inflammation. Increased use of anti-TNF antibodies including infliximab and adalimumab is due to the observed effectiveness of such drugs in corticosteroid nonresponsive cases (8).

A few studies have shown the effectiveness of anti-TNF therapy in the treatment of pulmonary and extra-pulmonary manifestations of sarcoidosis (9, 10). On the contrary, a great body of evidence supports the effectiveness of adalimumab in the treatment of refractory sarcoidosis (11, 12).

Our study result was in accordance with previous case series studies, confirming the effectiveness of adalimumab in the treatment of refractory sarcoidosis.

In spite of the responsiveness of refractory sarcoidosis to anti-TNF drugs, the definite indication, dosage, interval, and duration of the treatment remain to be established. Despite enough evidence for the effectiveness of adalimumab and infliximab in cases of refractory sarcoidosis, a trial with etanercept has not shown any considerable improvement in such cases (9).

Subcutaneous administration of adalimumab in contrast to the intravenous administration of infliximab makes the former a preferred choice of treatment. While adalimumab can be self-injected, infliximab can be administered only in a healthcare facility. Hence, for the convenience of the patient, we selected adalimumab for her treatment. Considering the high price and adverse effects of biological drugs, it is recommended to use them judiciously and less frequently. Similar to their use in other inflammatory disorders like Crohn’s disease, administration of anti-TNF drugs in sarcoidosis should focus on using an optimum dose for the induction and maintenance of remission. In this case, 40 mg subcutaneous injection of adalimumab was administered every 2 weeks, and there was still no clarity on how long the therapy will continue. As we attempted to increase the injection interval once, it led to the recurrence of symptoms. Eventually, we had to reduce the interval to 2 weeks again, which indicates that the decision to reduce the injection interval should not be rushed.

A well-defined anti-TNF therapeutic strategy for refractory sarcoidosis needs to be established for further investigation.
